# Synergistic Catalysis of Ruthenium Nanoparticles and Polyoxometalate Integrated Within Single UiO−66 Microcrystals for Boosting the Efficiency of Methyl Levulinate to γ-Valerolactone

**DOI:** 10.3389/fchem.2019.00042

**Published:** 2019-02-01

**Authors:** Xiaoxiong Cai, Qionghao Xu, Gaomei Tu, Yanghe Fu, Fumin Zhang, Weidong Zhu

**Affiliations:** Key Laboratory of the Ministry of Education for Advanced Catalysis Materials, Institute of Physical Chemistry, Zhejiang Normal University, Jinhua, China

**Keywords:** upgrading of biomass, synergistic catalysis, metal–organic framework, polyoxometalate, γ-valerolactone

## Abstract

The synthesis of heterogeneous cooperative catalysts in which two or more catalytically active components are spatially separated within a single material has generated considerable research efforts. The multiple functionalities of catalysts can significantly improve the efficiency of existing organic chemical transformations. Herein, we introduce ruthenium (Ru) nanoparticles (NPs) on the surfaces of a metal–organic framework pre-encapsulated with polyoxometalate silicotungstic acid (SiW) UiO−66 (University of Oslo [UiO]) and prepared a 2.0% Ru/11.7% SiW@UiO−66 porous hybrid using the impregnation method. The close synergistic effect of metal Ru NPs, SiW, and UiO-66 endow 2.0% Ru/11.7% SiW@UiO-66 with increased activity and stability for complete methyl levulinate (ML) conversion and exclusive γ-valerolactone (GVL) selectivity at mild conditions of 80°C and at a H_2_ pressure of 0.5 MPa. Effectively, this serves as a model reaction for the upgrading of biomass and outperforms the performances of the constituent parts and that of the physical mixture (SiW + Ru/UiO−66). The highly dispersed Ru NPs act as active centers for hydrogenation, while the SiW molecules possess Brønsted acidic sites that cooperatively promote the subsequent lactonization of MHV to generate GVL, and the UiO−66 crystal accelerates the mass transportation facilitated by its own porous structure with a large surface area.

## Introduction

Metal–organic frameworks (MOFs) are an emerging class of advanced functional materials that have generated tremendous research interest by virtue of their fascinating properties, such as the well-defined crystalline structures, large surface areas, tunable pore cavities, and abundant unsaturated metal sites in their scaffolds (Ma et al., 2009; Stock and Biswas, 2012; Furukawa et al., 2013; Chen et al., 2017; Jiao et al., 2017). Among the various MOFs, zirconium-based MOF UiO−66 (University of Oslo [UiO]), with a chemical formula of Zr_6_O_4_(OH)_4_(bdc)_6_ (bdc: 1,4-benzenedicarboxylate), is distinguished for its increased hydrothermal/chemical stability and good tolerance toward common organic solvents (Cavka et al., 2008; Kandiah et al., 2010). In addition, UiO−66 possesses a rigid three-dimensional cubic framework containing tetrahedral and octahedral cavities, with internal diameters that are approximately equal to 0.75 and 1.2 nm, respectively. These cavities are interconnected via microporous triangular pores with diameters of 0.6 nm, thus forming a high-porosity network. Thanks to these unique characteristics, the solid form of UiO−66 has been regarded as an ideal host matrix for encapsulation of metal nanoparticles (NPs) and guest molecules (Furukawa et al., 2014; Guo et al., 2014; Na et al., 2014; Yang et al., 2015a,b; Bai et al., 2016; Liu et al., 2016).

Production of biofuels and high-value biochemicals based on the utilization of biomass as starting feedstock has been identified as a sustainable strategy to mitigate the strong dependence on the rapidly diminishing fossil resources (Rojas-Buzo et al., 2018). Lignocellulosic biomass, including agricultural residues, wood, paper, and municipal solid waste, constitute the most abundant and biorenewable biomass on earth. Therefore, the transformation of lignocellulosic biomass to produce valuable biochemicals and biofuels has spurred intense efforts worldwide (Alonso et al., 2012; Zhang et al., 2017). Accordingly, γ-Valerolactone (GVL) is an intriguing platform molecule that originates from lignocellulosic biomass, and possesses tremendous potential for a variety of industrial applications (Serrano-Ruiz et al., 2011; Liguori et al., 2015; Zhang et al., 2016). GVL can be expediently generated via catalytic conversion of levulinic acid (LA) and its esters (Yan et al., 2015). Considering that LA is corrosive (*pKa* = 4.59) and that it easily induces the deactivation of catalysts owing to the leaching of active species, use of LA esters as starting reactants is a more advantageous option for biofuels and biochemical production (Wright and Palkovits, 2012). Considering methyl levulinate (ML) as an example, two steps are involved in the transformation of ML to GVL at relatively low-reaction temperatures: (a) the hydrogenation of ML to intermediate methyl-3-hydroxyvalerate (MHV), and (b) the successive transesterification of MHV to GVL, both of which are required to be catalyzed by metal NPs and acidic sites, respectively (Negahdar et al., 2017).

In the cohort of the various catalysts screened for the upgrade of LA and its esters to generate GVL (Du et al., 2011; Wright and Palkovits, 2012; Nadgeri et al., 2014; Tang et al., 2014; Ye et al., 2014; Kuwahara et al., 2015; Kadu et al., 2016; Winoto et al., 2016; Xiao et al., 2016; Albani et al., 2017; Hengst et al., 2017; Negahdar et al., 2017; Kondeboina et al., 2018; Li et al., 2018; Wang et al., 2018), precious metal ruthenium (Ru) NPs have been demonstrated to be the most active catalysts in liquid–phase catalytic reactions (Michel and Gallezot, 2015; Tan et al., 2015). Notably, the activation of the carbonyl group in the ML molecule by acid sites is the rate-determining step in the selective conversion of LA or ML to GVL. Thus, it has been reported that the conversion efficiency of LA or its esters could be enhanced over the Ru catalysts in the presence of the acid co-catalysts (Abdelrahman et al., 2014). For example, Galletti et al. evaluated the promotion effect of different solid acids on the Ru/C catalyst in the hydrogenation of LA to GVL, and found that the combination of Ru/C and resins Amberlyst A70 elicited the highest activity with a 99.9% GVL yield achieved at 70°C and at a H_2_ pressure of 3 MPa over a period of 3 h (Galletti et al., 2012). Barbaro et al. prepared a supported Ru catalyst with sulfonated resin as the support, and measured a 99.8% GVL yield at 70°C and at a H_2_ pressure of 0.5 MPa (Moreno-Marrodan and Barbaro, 2014). In our previous work, we reported the improved catalytic activity of Ru NPs supported on the acidified MOFs for the transformation of ML to GVL (Lin et al., 2017, 2018). These results manifest that the overall catalytic performance can be boosted considerably in the presence of the acid co-catalyst by significantly accelerating the sequential hydrogenation and lactonization steps in the upgrading process of LA and its esters.

**Scheme 1:**
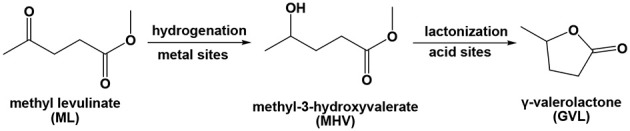
Tandem hydrogenation–lactonization of ML used to yield GVL in the presence of a metal/acid bifunctional catalyst.

Polyoxometalates (POMs) are a subset of anionic metal oxygen clusters of early transition metals that are extensively used as catalysts because of their facile tunable oxidation/redox and acid/base properties (Zeng et al., 2000; Du et al., 2014; Miras et al., 2014; Zhou et al., 2014; Zhang et al., 2015a,b; Buru et al., 2017, 2018). Using MOFs as the matrices to host POMs may elicit some benefits, such as the isolation of the POM molecules and the simultaneous improvement of substrate–POM accessibility (Miras et al., 2014; Zhang et al., 2015a,b; Buru et al., 2017, 2018). Notably, the cavities of UiO−66 are large enough to accommodate the POM molecules, while the apertures are small enough to prohibit the POM from leaching out of the pores. Based on the consideration of these facts, we report an approach that combines POM silicotungstic acid (H_4_SiW_12_O_40_·xH_2_O, abbreviated as SiW), Ru NPs, and MOF, in a UiO−66-based hybrid material, Ru/SiW@UiO−66, with a significantly increased activity and selectivity in the upgrade of ML to GVL relative to each of the constituent components, or their physical mixture. Specifically, in the synthesized catalyst Ru/SiW@UiO−66, the Ru NPs, which are distributed on the external surface of the UiO−66 crystals, can catalyze the hydrogenation of ML to form MHV owing to their activities and role for hydrogen activation and dissociation. While the SiW molecules which possess Brønsted acidic sites, which are encapsulated within the cavities of UiO−66, promote the subsequent lactonization of MHV to generate GVL owing to their excellent dealcoholization properties. In a cooperative manner, the UiO−66 crystal accelerates mass transportation which is facilitated by its own porous structure with a large surface area. Thus, the metal/SiW bifunctionalities within a single MOF crystal are anticipated to boost the tandem hydrogenation–lactonization reaction via a highly efficient synergistic catalysis manner (Scheme 1). To the best of our knowledge, this is an initial report on the preparation of MOF-based metal/acid bifunctional catalysts, in which the metal NPs and acidic site are spatially separated on the outer and internal spaces of the MOF, respectively, and their application in the upgrading of biomass.

## Experimental Section

Detailed information regarding the chemicals and methods can be found in Supporting Information.

### Catalyst Preparation

#### SiW@UiO−66

The UiO−66 crystals encapsulated SiW (SiW@UiO−66) which was synthesized by the one-pot hydrothermal method. In a typical synthesis, ZrCl_4_ (0.233 g, 1.0 mmol), H_2_BDC (0.166 g, 1.0 mmol), SiW (0.02 g, 6.95 × 10^−3^ mmol), and acetic acid (1 mL), were added to DMF (45 mL) during stirring to form a clear and colorless solution. After stirring for 30 min at 30°C, the solution was transferred into a Teflon-lined autoclave (100 mL) and was heated at 120°C for 24 h without stirring. The resulting white solid was collected by centrifugation and was washed with DMF and methanol three times. It was finally dried at 120°C for 12 h. For comparison, pure UiO−66 support was also synthesized following the same procedure as that described above but without the addition of SiW in the starting synthesis system.

#### Ru NPs

The Ru NPs were fabricated by a polyol reduction method (Zhao et al., 2016). In a typical procedure, 10.4 mg (0.5 mmol) RuCl_3_, and 58.0 mg (0.001 mmol) PVP (Ru/PVP molar ratio: 50:1) were dissolved in EG (10 mL). The reaction mixture was ultrasonicated for 5 min at 25°C. Subsequently, the solution was degassed at 80°C for 30 min with the use of flowing Ar in a three-necked flask. The solution was then heated to 180°C under flux and was maintained for 2 h in an inert Ar atmosphere. When the reaction was complete, acetone was added into the solution at room temperature, and the resulting cloudy black suspension was subjected to a centrifuge. The precipitated Ru NPs were then separated, collected, and redispersed in 80 mL ethanol (0.6 mmol/L).

#### Ru/SiW@UiO−66

To prepare the Ru/SiW@UiO−66 catalyst, pre-dried SiW@UiO−66 (0.1 g) was dispersed in a Ru NPs ethanol solution (33 mL) that had been processed by ultrasound for 15 min. After stirring at 40°C for 12 h, the resulting solid was collected by centrifugation at 8,000 rpm for 5 min, and was then dried under vacuum at 120°C for 4 h. For comparison, Ru NPs supported a UiO−66 sample (referred to as Ru/UiO−66), which was also prepared using the same method as that described above.

### Catalytic Activity Test

The catalytic upgrade of ML, a model compound of biomass, was performed in a Teflon-lined high-pressure reactor (50 mL, NS50–MP–LT–SS1–SV–BS, Anhui Kemi Machinery Technology Co. Ltd., Anhui, China) equipped with a gas inlet value and a sampling valve. The reactant ML (0.257 g, 1.98 mmol), pre-dried catalyst (50 mg, molar ratio of substrate to Ru NPs in the catalyst (S/C) was 200), and solvent H_2_O (15 mL) were added into the reactor. Prior to the reaction, the reactor was flushed with hydrogen six times without stirring. Once the desired temperature was reached (80°C), 0.5 MPa of hydrogen was introduced into the reactor, and the suspension was vigorously stirred at the constant speed of 980 rpm. This was considered as the onset of the reaction. During the reaction interval, the liquid samples were withdrawn regularly from the reactor and were analyzed by a Shimadzu GC−2014 gas chromatography with a flame ionization detector, with the use of a DB−5 capillary column. Upon completion of the reaction, the reactor was cooled down naturally to room temperature and was depressurized carefully. For the recyclability test, the spent Ru/SiW@UiO−66 was recovered by filtration, washed with ethanol three times, dried at 120°C, and was then subjected to the subsequent reaction cycle.

## Results and Discussion

### Catalyst Preparation and Characterization

**Scheme 2:**
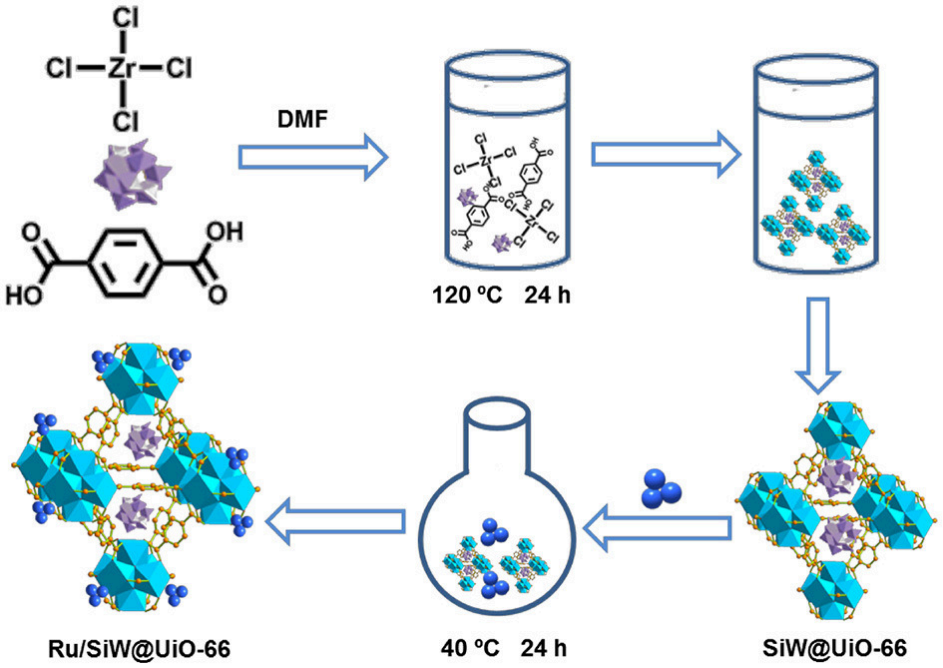
Schematic showing the preparation procedure of the Ru/SiW@UiO−66 catalyst. Purple polyhedra: SiW, dark blue ball: Ru NPs, sky blue framework: UiO−66.

Scheme 2 describes the preparation processes of the MOF-based metal/acid bifunctional hybrid by a facile two-step method. First, SiW molecules were confined within the UiO−66 cavities through the direct hydrothermal synthesis of UiO−66 in the presence of the preformed SiW. Subsequently, the pre-synthesized Ru NPs were loaded onto the external surfaces of the prepared SiW@UiO−66 by a conventional impregnation method. ICP–AES analyses revealed that the Ru and SiW contents within Ru/SiW@UiO−66 were 2.0 and 11.7% by weight, respectively (Table 1). These were very close to the nominal amounts added during the catalyst preparation procedure, thus implying that the *in-situ* encapsulation of SiW and subsequent immobilization of Ru NPs within the MOF was a feasible technique for catalyst preparation.

**Table 1:** Physicochemical properties of various catalysts investigated in this study.

**Catalyst**	***S_***BET***_*^**a**^ m^**2**^/g**	***V_***total***_*^**b**^ cm^**3**^/g**	***V_***micro***_*^**c**^ cm^**3**^/g**	**Acidity^**d**^ mmol/g**	**SiW^**e**^ %**	**Ru^**f**^ %**
UiO−66	1333	0.63	0.50	–	–	–
SiW@UiO−66	1200	0.54	0.44	–	–	–
Ru/UiO−66	1068	0.48	0.39	0.01	–	2.1
Ru/SiW@UiO-66	816	0.39	0.29	0.134	11.7	2.0
Spent Ru/SiW@UiO-66	811	0.39	0.29	0.131	11.6	1.9

a *BET specific area*.

b *Total pore volume*.

c *Microporous volume*.

d *Based on an acid–base titration method*.

e, f *Based on ICP–ASE analysis*.

**Figure 1:**
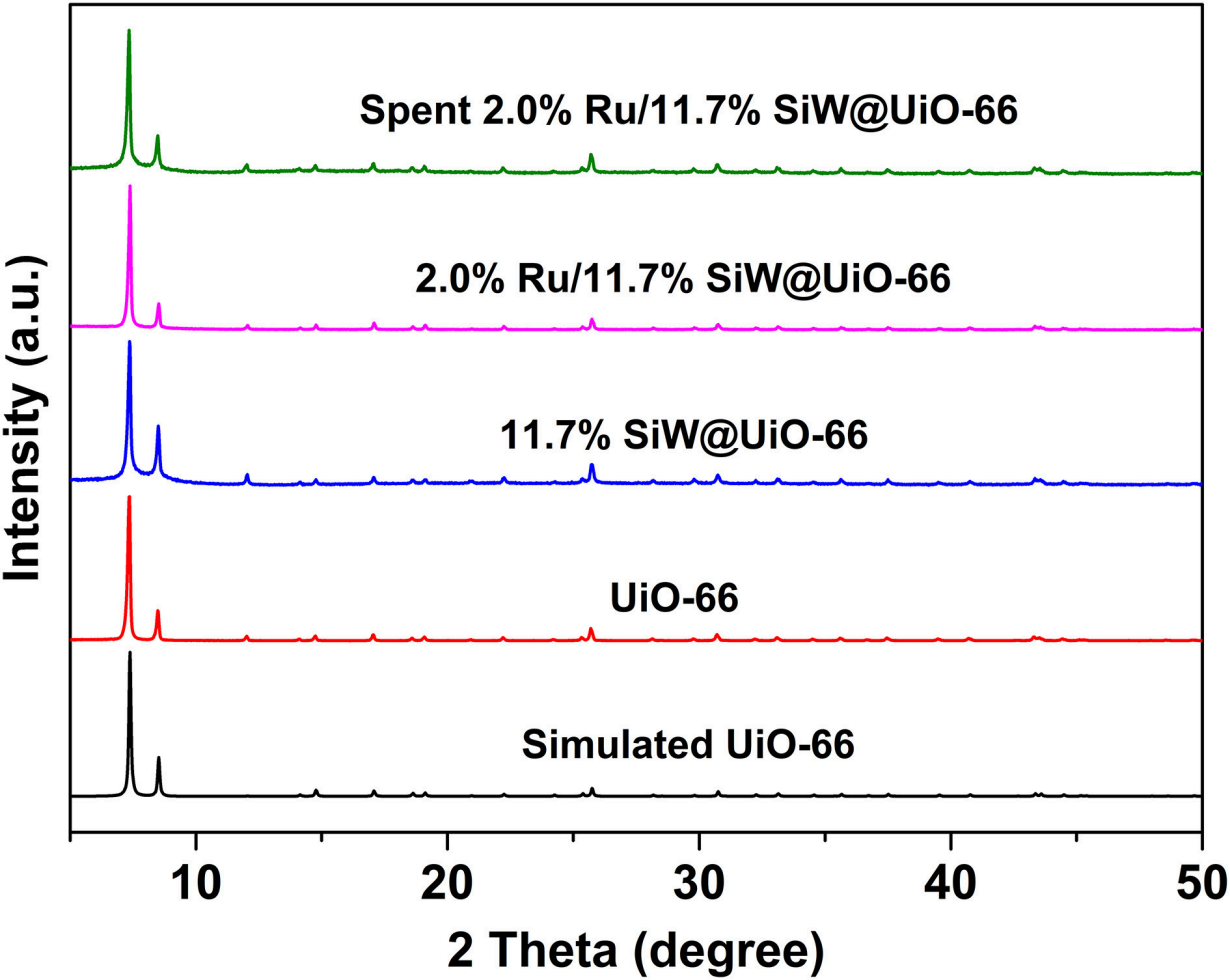
XRD patterns of various tested samples.

TGA results demonstrate that the prepared MOF and corresponding catalysts were stable up to 500°C (Figure S1 in Supporting Information). The XRD pattern of the synthesized UiO−66 support matches well the calculated pattern from crystal data (Figure 1) (Cavka et al., 2008; Kandiah et al., 2010). The crystal structure of 11.7% SiW@UiO−66, which remained almost unchanged compared to the prototype UiO−66, indicates that the addition of SiW molecules within UiO−66 hardly affected the crystalline structure of the pristine MOF (Yang et al., 2015b; Ullah et al., 2018). Moreover, no diffraction peaks associated with the SiW crystals were detected for 11.7% SiW@UiO−66, thus suggesting that the SiW nanoclusters were mainly confined within the MOF cavities. After the Ru NPs were introduced on SiW@UiO−66, the corresponding XRD pattern did not show any observable change probably owing to the very small Ru particle sizes and the low loading.

**Figure 2:**
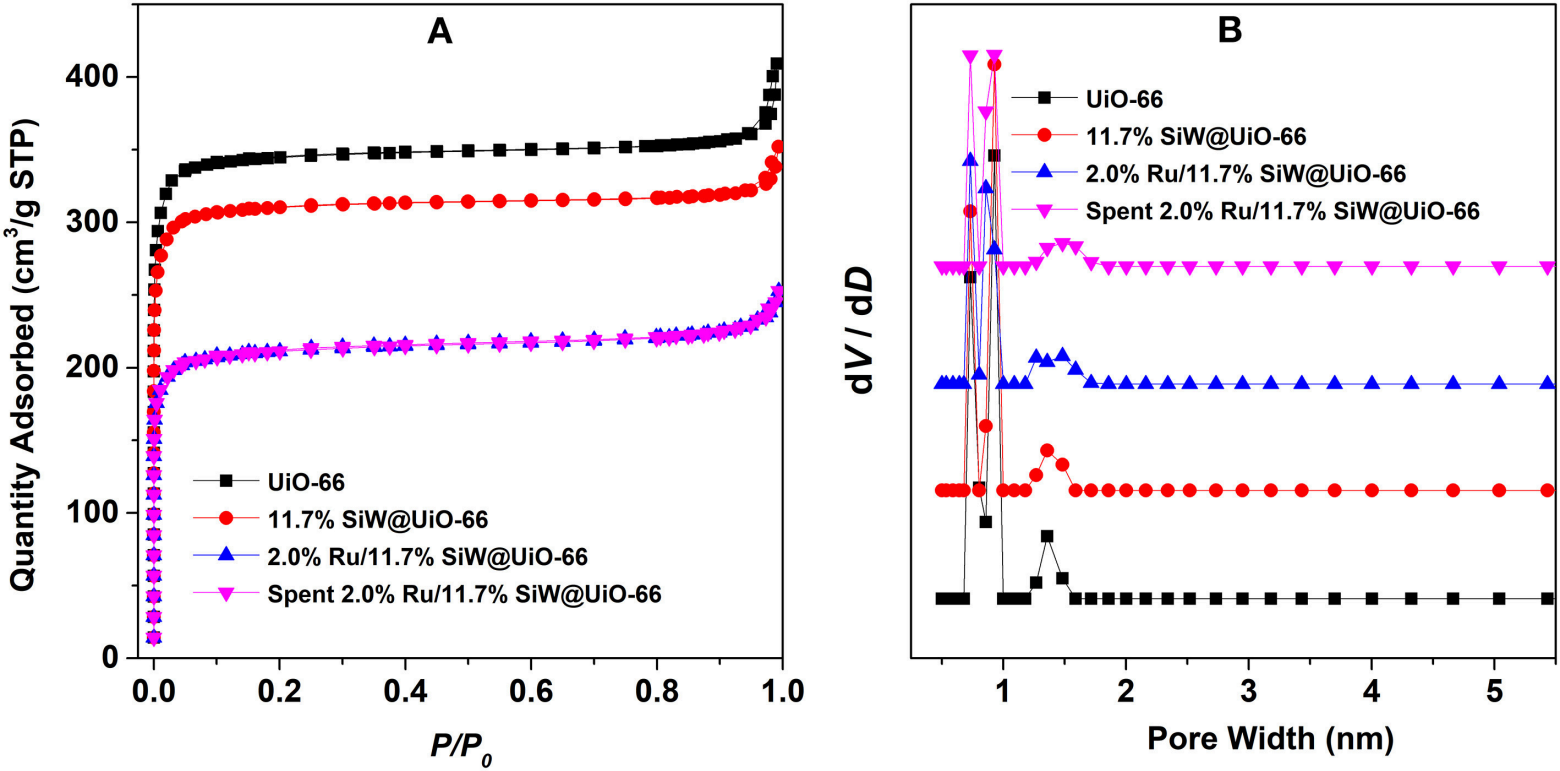
N_2_ adsorption isotherms at −196°C **(A)** and the corresponding pore size distribution plots **(B)** of the various tested samples.

N_2_ adsorption isotherms and the pore size distribution profiles of the various samples are shown in Figure 2. In addition, textural parameters as well as other physicochemical properties are summarized in Table 1. All the adsorption isotherms are type I curves according to the classification scheme of the International Union of Pure and Applied Chemistry, which verifies the inherit microporous structure of the prepared UiO−66-based samples (Cavka et al., 2008; Kandiah et al., 2010). Additionally, as listed in Table 1, the specific surface area (*S*_*BET*_) and total pore volume (*V*_*total*_) of UiO−66 are 1,333 m^2^/g and 0.63 cm^3^/g, respectively, which are much higher than the calculated value of perfect UiO−66 crystals, likely owing to missing linker defect sites that exist in the synthesized MOFs (Wu et al., 2013). As expected, both *S*_*BET*_ and *V*_*total*_ of SiW@UiO−66 decrease remarkably compared to the parent MOF. These changes are consistent with the SiW molecules incorporation in UiO−66. The pore size distribution of the various samples reveals two types of micropores: smaller pores with diameters in the range of 0.6–0.8 nm and larger pores in the range of 1.2–1.4 nm, thus confirming the presence of the two types of cages in these samples (Cavka et al., 2008; Kandiah et al., 2010; Ullah et al., 2018). After the Ru NPs were loaded onto SiW@UiO−66, the corresponding *S*_*BET*_ was remarkably reduced (816 m^2^/g). Meanwhile, the *V*_*total*_ also decreased from 0.54 to 0.39 cm^3^/g. The reductions in the surface area and pore volume were mainly attributed to the corresponding surface area and pore volume of SiW@UiO−66 which were occupied partially by the highly dispersed Ru NPs.

**Figure 3:**
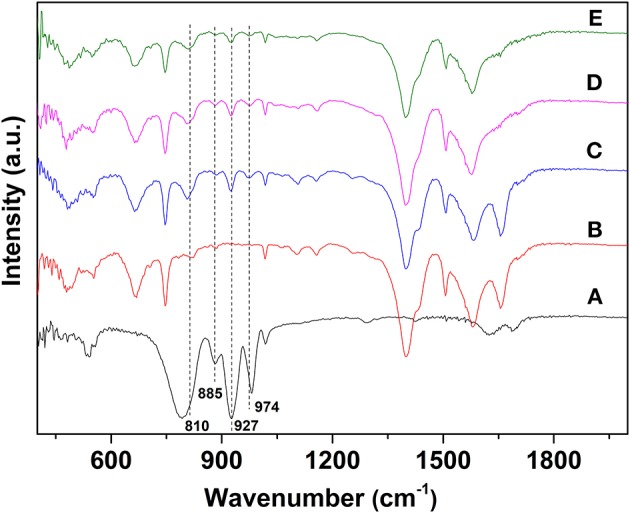
FTIR spectra of SiW **(A)**, UiO−66 **(B)**, 11.7% SiW@UiO−66 **(C)**, 2.0% Ru/11.7% SiW@UiO−66 **(D)**, and the used 2.0% Ru/11.7% SiW@UiO−66 catalyst **(E)**.

FTIR spectra have been used to analyze the bonding modes between UiO−66, SiW molecules, and Ru NPs (Figure 3). For SiW, the characteristic absorption peaks of the Keggin unit were detected at 974, 980, 922, and 810 cm^−1^, and are attributed to the v_as_(Si–O_a_), v_as_(W–O_d_), v_as_(W–O_b_-W), and v_as_(W–O_c_-W), respectively (Rajkumar and Ranga Rao, 2008). The FTIR spectrum of SiW@UiO−66 contains nearly all the characteristic peaks of SiW and UiO−66 with minor shifts for some bands, probably because of the confinement effect of the cavities of the UiO−66 matrix on the guest SiW molecules (Yang et al., 2015b; Ullah et al., 2018). Notably, the FTIR features associated with the SiW Keggin structure are well-preserved in the range of 800–1,000 cm^−1^ for the 2.0% Ru/11.7% SiW@UiO−66 sample (curve d in Figure 3).

**Figure 4:**
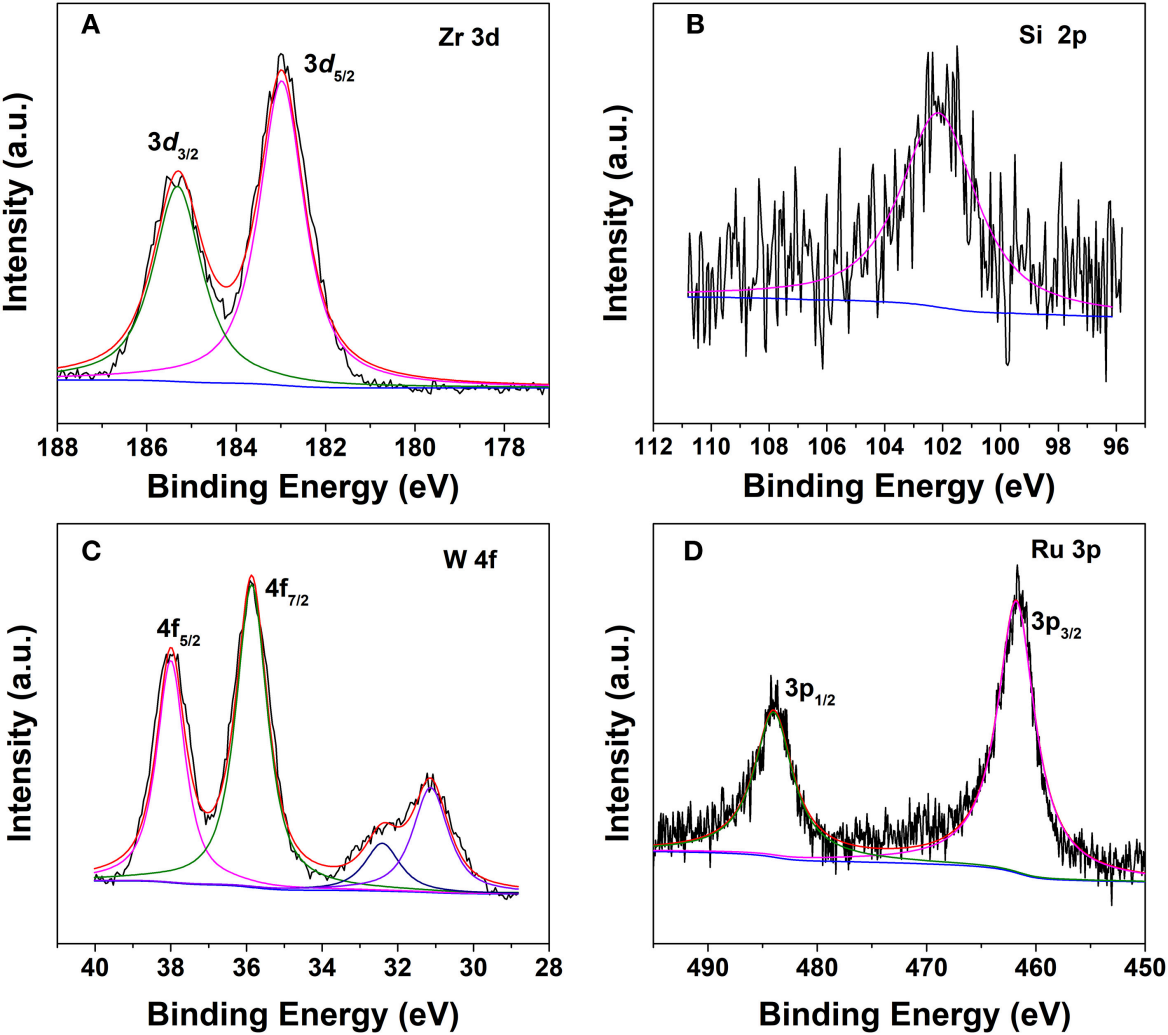
XPS spectra of 2.0% Ru/11.7% SiW@UiO−66. **(A)** Zr, **(B)** Si, **(C)** W, and **(D)** Ru.

Surface chemical composition and valence state of the various elements in the hybrid are characterized by the XPS technique. The Zr 3d spectrum can be deconvoluted into two peaks centered at 185.3 and 182.9 eV (Figure 4A), which are related to the electron binding energies of Zr 3d3/2 and Zr 3d5/2, respectively, similar to that of the pristine UiO−66 (Cavka et al., 2008). The peak for Si 2p in the Keggin structure of SiW was observed at 102.3 eV (Figure 4B) (Berry et al., 2009). For the tungsten species, two different chemical states were observed. The spin–orbit doublet with binding energies of 35.8 and 37.9 eV for W 4f7/2 and W 4f5/2, which account for ~70% of the total spectral area (Figure 4C). These values are typical for the presence of W^6+^, which is ascribed to SiW in the Keggin structure in the hybrid (Berry et al., 2009). A second doublet at 31.0 and 32.4 eV accounts for the remaining area, thus representing the partial decomposition of SiW within the MOF and the formation of an oxide of type WO_x_ in which W has an oxidation state lower than VI (Berry et al., 2009). Moreover, the sample exhibited Ru 3p bands at ca. 461.6 and 483.8 eV, which are the characteristic of zero-valent Ru species (Figure 4D).

**Figure 5:**
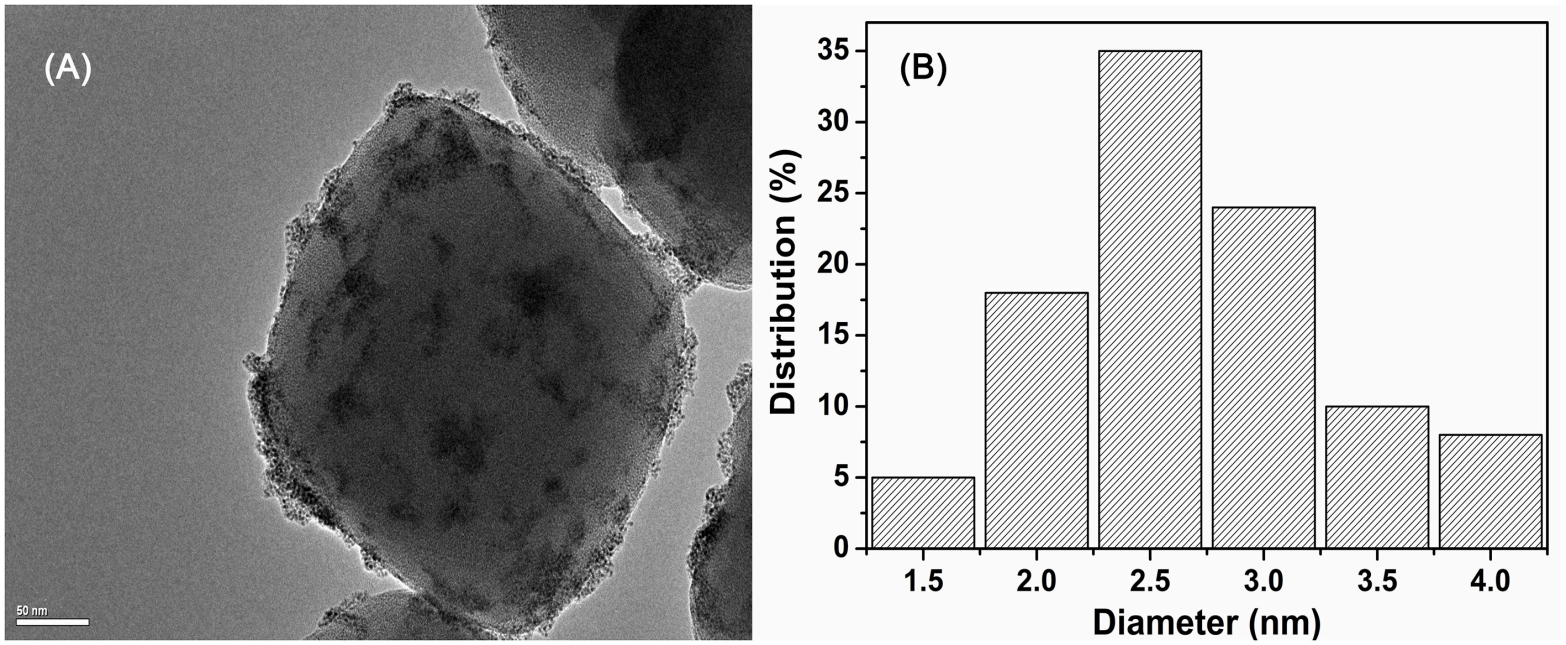
TEM image of 2.0% Ru/11.7% SiW@UiO−66 **(A)** and the corresponding size distribution plot of Ru NPs **(B)**.

The SEM image indicates that the pristine UiO−66 possesses well-defined octahedral microcrystals with an average crystal diameter of 150~200 nm (Figure S2). In the presence of SiW, the morphology of SiW@UiO−66 crystallites tends to be in the form of spherical particles, with the crystallite diameters of SiW@UiO−66 being very close to those of the UiO−66 (Figure S2). The shape evolutions are assumed to be originated from the binding of metal ions and SiW anions (Yang and Wang, 2018). As expected, both the size and morphology of Ru/UiO−66 and Ru/SiW@UiO−66 are almost the same as those for the supports (Figure S2). The TEM images demonstrate that the Ru NPs with a uniform size of 1.5–4 nm were highly distributed on both the surface of the UiO−66 and SiW@UiO−66 supports, as shown in Figure 5, Figure S3. The further EDX mapping also verified that the Zr, Ru, Si, W and were highly distributed within UiO-66 (Figure S4). The content of Brønsted acid sites in the SiW@UiO−66 samples were measured to be 0.134 mmol/g (Table 1). Even though the coordination of unsaturated Zr^4+^ sites within UiO−66 may serve as acidic sites (Cavka et al., 2008; Kandiah et al., 2010), their strengths are exceedingly weaker than those of the SiW sites. Thus, the measured acidity can be mainly attributed to the Brønsted acidic SiW sites confined within the UiO−66 frameworks. Therefore, the combined results of XRD, N_2_ adsorption, FTIR, XPS, SEM, TEM, and acid capacity measurements, confirm that the bifunctional 2.0% Ru/11.7% SiW@UiO−66 hybrid has been successfully prepared via the facile approach.

### Catalytic Studies

**Figure 6:**
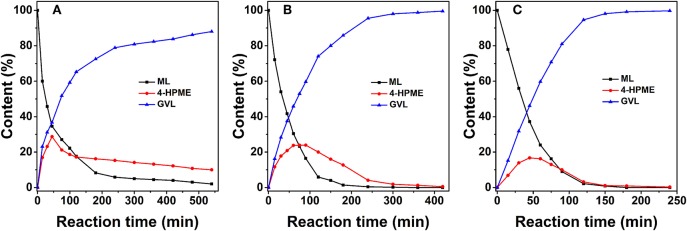
Concentration evolution profiles of ML, MHV, and GVL vs. time for 2.0% Ru/11.7% SiW@UiO−66 at 60°C **(A)**, 80°C **(B)**, and 100°C **(C)**. Reaction conditions: 0.257 g of ML, 50 mg of catalyst, 15 mL of H_2_O, and a H_2_ pressure of 0.5 MPa.

The composition, structure, and morphology of the 2.0% Ru/11.7% SiW@UiO−66 hybrid implies that it may be suitably used as an efficient bifunctional catalyst. Correspondingly, we evaluated its catalytic properties in the transformation of ML to GVL under mild reaction conditions using water as a green solvent. The influence of the reaction temperature on the reactant and product distribution was studied and the results were compared in Figure 6. Obviously, both the hydrogenation and the subsequent lactonization steps for the transformation of ML to GVL were significantly influenced by the reaction temperature, and MHV was produced as the intermediate. Furthermore, a stoichiometric equivalent amount of methanol to GVL was also obtained. As expected, both the conversion rate of ML and the generation rate of GVL were slow at 60°C. When the temperature increased to 100°C, the transformation rate increased distinctly. When ML was completely consumed, the concentration of MHV decreased gradually with a marginal increase in the GVL yield, thus indicating that the hydrogenation of ML to yield MHV was much easier than the transesterification of MHV to GVL at low temperatures (Lin et al., 2018). These results confirm that conversion of ML to GVL is a tandem reaction, and requires both metal and acid functionalities to work cooperatively (Nadgeri et al., 2014; Kuwahara et al., 2015). The transformation of the intermediate MHV to yield the final GVL product is probably the rate controlling step in this tandem reaction (Negahdar et al., 2017). Moreover, if Figure 6B is considered as an example, the transformation is shown to be accompanied by a rapid decrease in the concentration of ML and an increase in MHV in the first 75 min. As the reaction progressed, the intramolecular de-alcoholization of MHV to GVL proceeded rapidly. These results consistently prove that the ML-to-GVL over the 2.0% Ru/11.7% SiW@UiO−66 catalyst followed the procedure shown in Scheme 1. Importantly, it should be stated that 100% ML conversion with exclusive selectivity for GVL were achieved over the 2.0% Ru/11.7% SiW@UiO−66 catalyst within 360 min when the reaction was performed at 80°C and at a hydrogen pressure of 0.5 MPa. Additionally, no further by-product was formed even when the reaction time was extended to 520 min.

**Figure 7:**
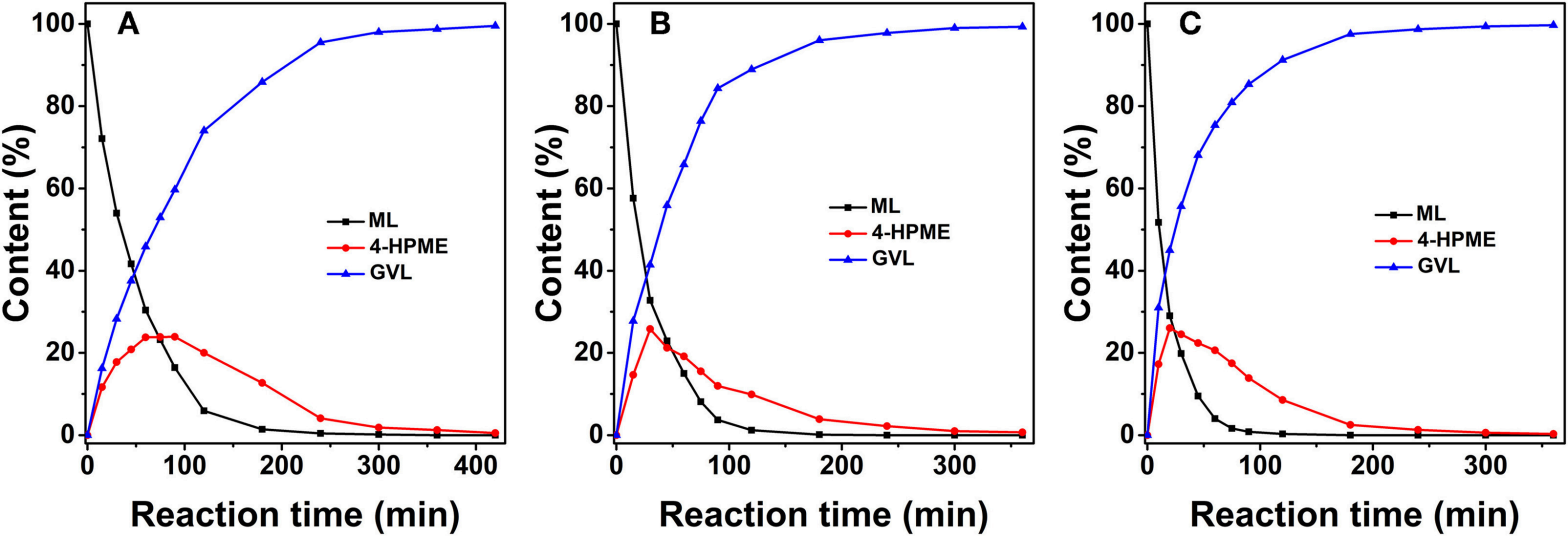
Concentration evolution profiles of ML, MHV, and GVL vs. time over 2.0% Ru/11.7% SiW@UiO−66 at a H_2_ pressure of 0.5 MPa **(A)**, 1.0 MPa **(B)**, and 1.5 MPa **(C)**. Reaction conditions: 0.257 g of ML, 50 mg of catalyst, 15 mL of H_2_O, and a temperature of 80°C.

The influence of the H_2_ pressure on the reactant and product distributions as a function of reaction time over 2.0% Ru/11.7% SiW@UiO−66 is presented in Figure 7. As expected, the dissolved hydrogen in the reaction solution would increase when the H_2_ pressure in the reactor increases. Therefore, the hydrogenation of ML to MHV would be remarkably enhanced. Moreover, the lactonization of MHV to GVL is proportional with respect to the concentration of MHV, and can be accelerated by the Brønsted acid sites in the catalyst (Hao et al., 2018). Therefore, the overall catalytic activity and selectivity of ML to GVL was essentially improved as the H_2_ pressure increased.

**Figure 8:**
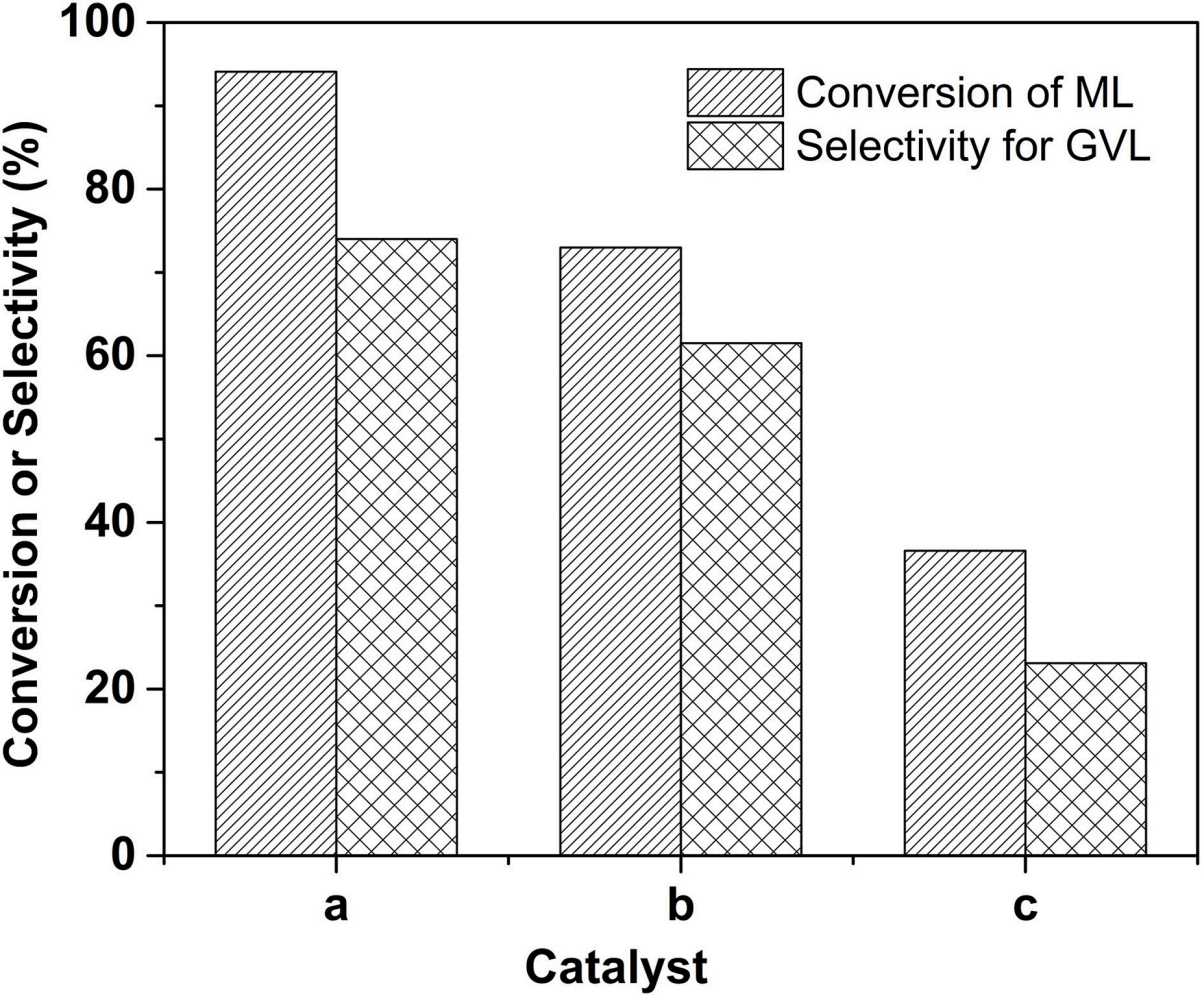
Comparison of conversion and selectivity for various catalysts. Reaction conditions: 0.257 g of ML, 50 mg of 2.0% Ru/11.7% SiW@UiO−66 for case a; 5 mg of SiW + 50 mg of 2.0% Ru/UiO−66 for case b; 50 mg of 2.0% Ru/UiO−66 for case c, 15 mL of H_2_O, 80°C, 0.5 MPa H_2_, during a 120 min reaction period.

To demonstrate the indispensable role of acid sites incorporated within the catalyst, the concentration evolution profiles over two controlled catalysts were also monitored under the identical reaction conditions, as displayed in Figures S5,S6. Overall, the catalytic activities over these two catalysts are lower than that of the 2.0% Ru/11.7% SiW@UiO−66 catalyst. For a more intuitive comparison, the catalytic results over 2.0% Ru/11.7% SiW@UiO−66, 2.0% Ru/UiO−66, and physical mixture of SiW and 2.0% Ru/UiO−66 under 80°C and 0.5 MP H_2_ pressure for a duration of 120 min were extracted (Figure 8). Notably, in the presence of 2.0% Ru/11.7% SiW@UiO−66, satisfactory results for the sequential hydrogenation and the subsequent lactonization reaction were achieved, with a 94.1% conversion of ML and a 74% selectivity for the GVL product. In contrast, in the presence of the 2.0% Ru/UiO−66 catalyst, a rather low catalytic activity and selectivity were obtained when the reaction was performed under the same conditions. This is probably owing to the lower acid content of the UiO−66 as compared to that of the SiW@UiO−66 support (see Table 1). It has been reported that the Brønsted acid sites were likely used to cooperatively catalyze sequential hydrogenation and lactonization with the active metal counterpart (Lin et al., 2017). To test this hypothesis, the native 2.0% Ru/UiO−66 was physically mixed with SiW that contained Brønsted acid sites. This reaction elicited moderate improvement regarding the ML-to-GVL activity compared to 2.0% Ru/11.7% SiW@UiO−66. We deduced that the (1) Brønsted acid sites that originated from the SiW molecules were efficient for C–O scission and could dramatically accelerate the transesterification of MHV to generate GVL, and (2) the acids sites should be in close proximity to metal sites for synergetic catalysis. In addition to the activation of the lactonization step, the Brønsted acid sites also likely contributed to the promotion of ML hydrogenation, thus confirming that 2.0% Ru/11.7% SiW@UiO−66 improved the activity in the upgrade of ML to GVL based on a bifunctional way.

Furthermore, representative results for catalytic conversion of ML to GVL by heterogeneous catalysts reported in the literature were compared with our catalyst, and these data were listed in Table S1. Due to the different reaction conditions for these works, it is difficult to compare the catalytic activity directly. Considering that the catalytic reaction was performed under a relative mild condition in the current study, and the 2.0% Ru/11.7% SiW@UiO-66 catalyst exhibited a relatively higher catalytic performance, the comparison tentative demonstrates that the developed catalyst is among the best of candidates ever reported on Ru-based heterogeneous catalysts as far as we know.

**Figure 9:**
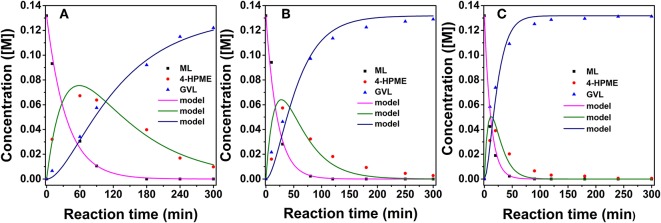
Experimental data fitting at 40°C **(A)**, 60°C **(B)**, and 80°C **(C)** for 2.0% Ru/11.7% SiW@UiO−66. Reaction conditions: 0.257 g of ML, 50 mg of catalyst, 15 mL of H_2_O, and an H_2_ pressure of 2.0 MPa.

**Figure 10:**
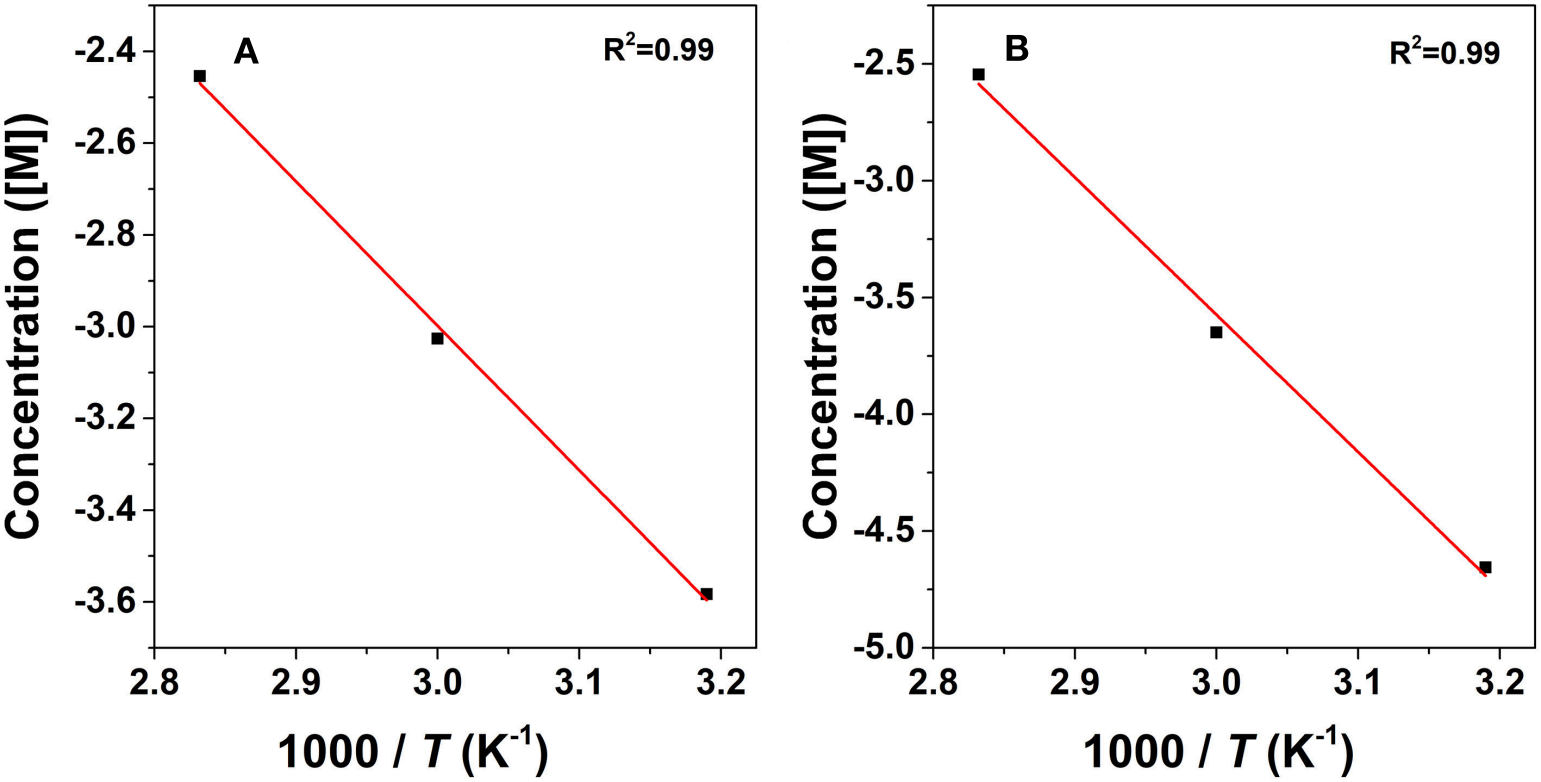
Arrhenius plots for the conversion of ML to GVL: hydrogenation step **(A)** and transesterification step **(B)**.

To elucidate the efficiency of 2.0% Ru/11.7% SiW@UiO−66 as a bifunctional catalyst, kinetic studies were performed when the internal and external transport limitations were eliminated according to a similar procedure described in our previous work (Lin et al., 2018). As demonstrated in Figure 9, the evolution of reactant concentrations at the evaluated temperatures can be best fitted using the following equations,

(1) $$\begin{array}{rcl} \frac{dC_{ML}}{dt} & = & -k_{1}C_{ML} \end{array}$$

(2) $$\begin{array}{rcl} \frac{dC_{MHV}}{dt} & = & k_{1}C_{ML}-k_{2}C_{MHV} \end{array}$$

(3) $$\begin{array}{rcl} \frac{dC_{GVL}}{dt} & = & k_{2}C_{MHV} \end{array}$$

where *C*_*i*_ represents the concentration of the component and *k*_*i*_is the reaction rate coefficient. As listed in Table 2, the rate constant of the lactonization step is smaller than that of the hydrogenation step, thus indicating that lactonization is the rate-determining step and controls the overall reaction rate. Subsequently, the activation energy barrier is calculated according to the Arrhenius equation: *k* = A × exp(–*Ea*/*RT*) (*k*: the aforementioned reaction rate coefficient, A: pre-exponential factor, *Ea*: activation energy, *R*: gas constant, and *T*: reaction temperature). Figure 10 shows the linear regression of ln*k* vs. 1/*T* for which the estimated slope is –*Ea/R*. The estimated activation energy for hydrogenation is 26.1 kJ/mol and that for lactonization is 48.8 kJ/mol (Table 2), which are much lower than the previously reported values for the aqueous hydrogenation of ML to GVL over Ru/C (41 kJ/mol for the hydrogenation and 50 kJ/mol for the lactonization) (Negahdar et al., 2017). This further indicates the advantages of integrating metal and acid sites within single MOF nanocrocrystals, that is, upon the intrinsic cooperation of the active sites, the energy barrier is reduced, thus facilitating the catalytic conversion of ML into GVL.

**Table 2:** Kinetic parameters of 2.0% Ru/11.7% SiW@UiO−66 in the upgrade of ML to GVL.

***T* (^**°**^C)**	**40**	**60**	**80**	***Ea* (KJ/mol)**	***R*^**2**^**
*k_1_* (10^−2^/min)	2.78	4.85	8.60	26.1	0.99
*k_2_* (10^−2^/min)	0.95	2.60	7.85	48.8	0.99

**Figure 11:**
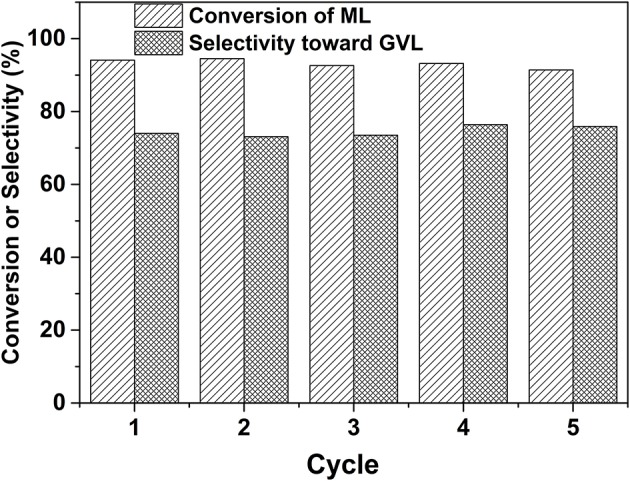
Durability test of 2.0% Ru/11.7% SiW@UiO−66 for the conversion of ML to GVL. Reaction conditions: 0.257 g of ML, 50 mg of catalyst, 15 mL of H_2_O, 80°C, an H_2_ pressure of 0.5 MPa H_2_, within a reaction period of 120 min.

The durability of the prepared 2.0% Ru/11.7% SiW@UiO−66 was further evaluated. As shown in Figure 11, both ML conversion and GVL selectivity were well-maintained after the catalyst was repetitively used for five successive cycles, thus demonstrating its excellent reusability capacity. Regarding the recovered catalyst, no changes in the crystalline structure, composition, or textural properties, have been noted as compared to the fresh one (Figures 1–3, Figure S7), thus confirming its excellent stability. Moreover, based on the ICP–AES and TEM results of the catalyst after usage (Figure S8), 2.0% Ru/11.7% SiW@UiO−66 exhibited good resistance to the leaching and sintering of POM species and Ru NPs, which was probably due to the fact that SiW molecules were well-confined by the MOF cavities and Ru NPs embedded and stabilized by the local defect sites of UiO−66 (Miras et al., 2014). These results indicate that the developed 2.0% Ru/11.7% SiW@UiO−66 may be a promising catalyst for other reactions that require both metal and acid sites to work cooperatively.

## Conclusions

In summary, we designed and prepared a MOF-based metal and acid bifunctional hybrid as an efficient heterogeneous catalyst for the upgrade of ML to produce valuable GVL under mild reaction conditions. The developed 2.0% Ru/11.7% SiW@UiO−66 catalyst exhibited high catalytic activity and high selectivity in the successive hydrogenation and lactonization steps, and it was proved that it could be repeatedly used for five times without any loss in activity and selectivity. The increased catalytic performance of the 2.0% Ru/11.7% SiW@UiO−66 catalyst was principally originated in the cooperative effect between the Ru NPs and Brønsted acid sites. These sites were appropriately spatially separated within a single UiO−66 crystal, and provided the active sites where the hybrid could catalyze the hydrogenation of ML to produce the intermediate MHV and the successive intramolecular de-alcoholization to form GVL. A kinetics study further demonstrated that the encapsulation of the SiW molecules within the UiO−66 cavities was favorable for the hydrogenation and lactonization stepsa property likely to be attributed to their Brønsted acidity properties.

## Author Contributions

XC conceived the idea, proposed the strategy, designed and performed the experiment, analyzed the results, and wrote the manuscript. QX and YF helped with the catalyst evaluation and corresponding data analysis and discussions. GT, FZ, and WZ supervised the project, helped design the experiments, evaluated the data, and wrote the manuscript. The results of the manuscript were discussed by all authors.

### Conflict of Interest Statement

The authors declare that the research was conducted in the absence of any commercial or financial relationships that could be construed as a potential conflict of interest.

## References

[B1] Abdelrahman O. A. Heyden A. Bond J. Q. (2014). Analysis of kinetics and reaction pathways in the aqueous-phase hydrogenation of levulinic acid to form γ-valerolactone over Ru/C. ACS Catal. 4, 1171–1181. 10.1021/cs401177p

[B2] Albani D. Li Q. Vilé G. Mitchell S. Almora-Barrios N. Witte P. T. (2017). Interfacial acidity in ligand-modified ruthenium nanoparticles boosts the hydrogenation of levulinic acid to γ-valerolactone. Green Chem. 19, 2361–2370. 10.1039/C6GC02586B

[B3] Alonso D. M. Wettstein S. G. Dumesic J. A. (2012). Bimetallic catalysts for upgrading of biomass to fuels and chemicals. Chem. Soc. Rev. 41, 8075–8098. 10.1039/c2cs35188a 22872312

[B4] Bai Y. Dou Y. Xie L. H. Rutledge W. Li J. R. Zhou H. C. (2016). Zr-based metal-organic frameworks: design, synthesis, structure, and applications. Chem. Soc. Rev. 45, 2327–2367. 10.1039/C5CS00837A 26886869

[B5] Berry F. J. Derrick G. R. Marco J. F. Mortimer M. (2009). Silica-supported silicotungstic acid: a study by X-ray photoelectron spectroscopy. Mater. Chem. Phys. 114, 1000–1003. 10.1016/j.matchemphys.2008.11.003

[B6] Buru C. T. Li P. Mehdi B. L. Dohnalkova A. Platero-Prats A. E. Browning N. D. (2017). Adsorption of a catalytically accessible polyoxometalate in a mesoporous channel-type metal–organic framework. Chem. Mater. 29, 5174–5181. 10.1021/acs.chemmater.7b00750

[B7] Buru C. T. Platero-Prats A. E. Chica D. G. Kanatzidis M. G. Chapman K. W. Farha O. K. (2018). Thermally induced migration of a polyoxometalate within a metal–organic framework and its catalytic effects. J. Mater. Chem. A 6, 7389–7394. 10.1039/C8TA02562B

[B8] Cavka J. H. Jakobsen S. Olsbye U. Guillou N. Lamberti C. Bordiga S.. (2008). A new zirconium inorganic building brick forming metal organic frameworks with exceptional stability. J. Am. Chem. Soc. 130, 13850–13851. 10.1021/ja8057953 18817383

[B9] Chen L. Luque R. Li Y. (2017). Controllable design of tunable nanostructures inside metal-organic frameworks. Chem. Soc. Rev. 46, 4614–4630. 10.1039/C6CS00537C 28516998

[B10] Du D. Y. Qin J. S. Li S. L. Su Z. M. Lan Y. Q. (2014). Recent advances in porous polyoxometalate-based metal–organic framework materials. Chem. Soc. Rev. 43, 4615–4632. 10.1039/C3CS60404G 24676127

[B11] Du X. L. Bi Q. Y. Liu Y. M. Cao Y. Fan K. N. (2011). Conversion of biomass-derived levulinate and formate esters into γ-valerolactone over supported gold catalysts. ChemSusChem 4, 1838–1843. 10.1002/cssc.201100483 22105964

[B12] Furukawa H. Cordova K. E. O'Keeffe M. Yaghi O. M. (2013). The chemistry and applications of metal-organic frameworks. Science 341:1230444 10.1126/science.1230444 23990564

[B13] Furukawa H. Gándara F. Zhang Y. B. Jiang J. Queen W. L. Hudson M. R.. (2014). Water adsorption in porous metal-organic frameworks and related materials. J. Am. Chem. Soc. 136, 4369–4381. 10.1021/ja500330a 24588307

[B14] Galletti A. M. R. Antonetti C. De Luise V. Martinelli M. (2012). A sustainable process for the production of γ-valerolactone by hydrogenation of biomass-derived levulinic acid. Green Chem. 14, 688–694. 10.1039/c2gc15872h

[B15] Guo Z. Y. Xiao C. X. Maligal-Ganesh R. V. Zhou L. Goh T. W. Li X. L. (2014). Pt Nanoclusters confined within metal organic framework cavities for chemoselective cinnamaldehyde hydrogenation. ACS Catal. 4, 1340–1348. 10.1021/cs400982n

[B16] Hao P. Schwartz D. K. Medlin J. W. (2018). Phosphonic acid promotion of supported Pd catalysts for low temperature vanillin hydrodeoxygenation in ethanol. Appl. Catal. A Gen. 561, 1–6. 10.1016/j.apcata.2018.05.008

[B17] Hengst K. Ligthart D. A. J. M. Doronkin D. E. Walter K. M. Kleist W. Hensen E. J. M. (2017). Continuous synthesis of γ-valerolactone in a trickle-bed reactor over supported nickel catalysts. Ind. Eng. Chem. Res. 56, 2680–2689. 10.1021/acs.iecr.6b03493

[B18] Jiao L. Wang Y. Jiang H. L. Xu Q. (2017). Metal-organic frameworks as platforms for catalytic applications. Adv. Mater. 30:e1703663. 10.1002/adma.201703663 29178384

[B19] Kadu B. S. Hengne A. M. Biradar N. S. Rode C. V. Chikate R. C. (2016). Reductive cyclization of levulinic acid to γ-valerolactone over non-noble bimetallic nanocomposite. Ind. Eng. Chem. Res. 55, 13032–13039. 10.1021/acs.iecr.6b03900

[B20] Kandiah M. Nilsen M. H. Usseglio S. Jakobsen S. Olsbye U. Tilset M. (2010). Synthesis and stability of tagged UiO-66 Zr-MOFs. Chem. Mater. 22, 6632–6640. 10.1021/cm102601v

[B21] Kondeboina M. Enumula S. S. Gurram V. R. B. Chada R. R. Burri D. R. Kamaraju S. R. R. (2018). Selective hydrogenation of biomass-derived ethyl levulinate to γ-valerolactone over supported Co catalysts in continuous process at atmospheric pressure. J. Ind. Eng. Chem. 61, 227–235. 10.1016/j.jiec.2017.12.020

[B22] Kuwahara Y. Magatani Y. Yamashita H. (2015). Ru nanoparticles confined in Zr-containing spherical mesoporous silica containers for hydrogenation of levulinic acid and its esters into γ-valerolactone at ambient conditions. Catal. Today 258, 262–269. 10.1016/j.cattod.2015.01.015

[B23] Li F. Li Z. France L. J. Mu J. Song C. Chen Y. (2018). Highly efficient transfer hydrogenation of levulinate esters to γ-valerolactone over basic zirconium carbonate. Ind. Eng. Chem. Res. 57, 10126–10136. 10.1021/acs.iecr.8b00712

[B24] Liguori F. Moreno-Marrodan C. Barbaro P. (2015). Environmentally friendly synthesis of γ-valerolactone by direct catalytic conversion of renewable sources. ACS Catal. 5, 1882–1894. 10.1021/cs501922e

[B25] Lin Z. Cai X. Fu Y. Zhu W. Zhang F. (2017). Cascade catalytic hydrogenation-cyclization of methyl levulinate to form γ-valerolactone over Ru nanoparticles supported on a sulfonic acid-functionalized UiO-66 catalyst. RSC Adv. 7, 44082–44088. 10.1039/C7RA06293A

[B26] Lin Z. Luo M. Zhang Y. Wu X. Fu Y. Zhang F. (2018). Coupling Ru nanoparticles and sulfonic acid moieties on single MIL-101 microcrystals for upgrading methyl levulinate into γ-valerolactone. Appl. Catal. A Gen. 563, 54–63. 10.1016/j.apcata.2018.06.027

[B27] Liu H. Chang L. Bai C. Chen L. Luque R. Li Y. (2016). Controllable encapsulation of “clean” metal clusters within mofs through kinetic modulation: towards advanced heterogeneous nanocatalysts. Angew. Chem. Int. Ed. 55, 5019–5023. 10.1002/anie.201511009 26970412 PMC5069584

[B28] Ma L. Abney C. Lin W. (2009). Enantioselective catalysis with homochiral metal-organic frameworks. Chem. Soc. Rev. 38, 1248–1256. 10.1039/b807083k 19384436

[B29] Michel C. Gallezot P. (2015). Why is ruthenium an efficient catalyst for the aqueous-phase hydrogenation of biosourced carbonyl compounds? ACS Catal. 5, 4130–4132. 10.1021/acscatal.5b00707

[B30] Miras H. N. Vilà-Nadal L. Cronin L. (2014). Polyoxometalate based open-frameworks (POM-OFs). Chem. Soc. Rev. 43, 5679–5699. 10.1039/C4CS00097H 25005364

[B31] Moreno-Marrodan C. Barbaro P. (2014). Energy efficient continuous production of γ-valerolactone by bifunctional metal/acid catalysis in one pot. Green Chem. 16, 3434–3438. 10.1039/c4gc00298a

[B32] Na K. Choi K. M. Yaghi O. M. Somorjai G. A. (2014). Metal nanocrystals embedded in single nanocrystals of MOFs give unusual selectivity as heterogeneous catalysts. NANO Lett. 14, 5979–5983. 10.1021/nl503007h 25198135

[B33] Nadgeri J. M. Hiyoshi N. Yamaguchi A. Sato O. Shirai M. (2014). Liquid phase hydrogenation of methyl levulinate over the mixture of supported ruthenium catalyst and zeolite in water. Appl. Catal. A Gen. 470, 215–220. 10.1016/j.apcata.2013.10.059

[B34] Negahdar L. Al-Shaal M. G. Holzhäuser F. J. Palkovits R. (2017). Kinetic analysis of the catalytic hydrogenation of alkyl levulinates to γ-valerolactone. Chem. Eng. Sci. 158, 545–551. 10.1016/j.ces.2016.11.007

[B35] Rajkumar T. Ranga Rao G. (2008). Synthesis and characterization of hybrid molecular material prepared by ionic liquid and silicotungstic acid. Mater. Chem. Phys. 112, 853–857. 10.1016/j.matchemphys.2008.06.046

[B36] Rojas-Buzo S. García-García P. Corma A. (2018). Catalytic transfer hydrogenation of biomass-derived carbonyls over hafnium-based metal-organic frameworks. ChemSusChem 11, 432–438. 10.1002/cssc.201701708 29139603

[B37] Serrano-Ruiz J. C. Luque R. Sepúlveda-Escribano A. (2011). Transformations of biomass-derived platform molecules: from high added-value chemicals to fuels via aqueous-phase processing. Chem. Soc. Rev. 40, 5266–5281. 10.1039/c1cs15131b 21713268

[B38] Stock N. Biswas S. (2012). Synthesis of metal-organic frameworks (MOFs): routes to various MOF topologies, morphologies, and composites. Chem. Rev. 112, 933–969. 10.1021/cr200304e 22098087

[B39] Tan J. Cui J. Deng T. Cui X. Ding G. Zhu Y. (2015). Water-promoted hydrogenation of levulinic acid to γ-valerolactone on supported ruthenium catalyst. ChemCatChem 7, 508–512. 10.1002/cctc.201402834

[B40] Tang X. Chen H. Hu L. Hao W. Sun Y. Zeng X. (2014). Conversion of biomass to γ-valerolactone by catalytic transfer hydrogenation of ethyl levulinate over metal hydroxides. Appl. Catal. B Environ. 147, 827–834. 10.1016/j.apcatb.2013.10.021

[B41] Ullah L. Zhao G. Xu Z. He H. Usman M. Zhang S. (2018). 12-Tungstophosphoric acid niched in Zr-based metal-organic framework: a stable and efficient catalyst for Friedel-Crafts acylation. Sci. China Chem. 61, 402–411. 10.1007/s11426-017-9182-0

[B42] Wang Y. Rong Z. Wang Y. Wang T. Du Q. Wang Y. (2018). Graphene-based metal/acid bifunctional catalyst for the conversion of levulinic acid to γ-valerolactone. ACS Sustain. Chem. Eng. 5, 1538–1548. 10.1021/acssuschemeng.6b02244

[B43] Winoto H. P. Ahn B. S. Jae J. (2016). Production of γ-valerolactone from furfural by a single-step process using Sn-Al-Beta zeolites: optimizing the catalyst acid properties and process conditions. J. Ind. Eng. Chem. 40, 62–71. 10.1016/j.jiec.2016.06.007

[B44] Wright W. R. Palkovits R. (2012). Development of heterogeneous catalysts for the conversion of levulinic acid to γ-valerolactone. ChemSusChem 5, 1657–1667. 10.1002/cssc.201200111 22890968

[B45] Wu H. Chua Y. S. Krungleviciute V. Tyagi M. Chen P. Yildirim T.. (2013). Unusual and highly tunable missing-linker defects in zirconium metal–organic framework UiO-66 and their important effects on gas adsorption. J. Am. Chem. Soc. 135, 10525–10532. 10.1021/ja404514r 23808838

[B46] Xiao C. Goh T. W. Qi Z. Goes S. Brashler K. Perez C. (2016). Conversion of levulinic acid to γ-valerolactone over few-layer graphene-supported ruthenium catalysts. ACS Catal. 6, 593–599. 10.1021/acscatal.5b02673

[B47] Yan K. Yang Y. Chai J. Lu Y. (2015). Catalytic reactions of γ-valerolactone: a platform to fuels and value-added chemicals. Appl. Catal. B Environ. 179, 292–304. 10.1016/j.apcatb.2015.04.030

[B48] Yang D. Odoh S. O. Wang T. C. Farha O. K. Hupp J. T. Cramer C. J.. (2015a). Metal-organic framework nodes as nearly ideal supports for molecular catalysts: NU-1000-and UiO-66-supported iridium complexes. J. Am. Chem. Soc. 137, 7391–7396. 10.1021/jacs.5b02956 25990021

[B49] Yang H. Wang X. (2018). Secondary-component incorporated hollow MOFs and derivatives for catalytic and energy-related applications. Adv. Mater. 10.1002/adma.201800743. [Epub ahead of print]. 30039881

[B50] Yang X. L. Qiao L. M. Dai W. L. (2015b). Phosphotungstic acid encapsulated in metal-organic framework UiO-66: An effective catalyst for the selective oxidation of cyclopentene to glutaraldehyde. Micropor. Mesopor. Mater. 211, 73–81. 10.1016/j.micromeso.2015.02.035

[B51] Ye F. Zhang D. Xue T. Wang Y. Guan Y. (2014). Enhanced hydrogenation of ethyl levulinate by Pd-AC doped with Nb_2_O_5_. Green Chem. 16, 3951–3957. 10.1039/C4GC00972J

[B52] Zeng H. Newkome G. R. Hill C. L. (2000). Poly (polyoxometalate) dendrimers: molecular prototypes of new catalytic materials. Angew. Chem. Int. Ed. 39, 1771–1774. 10.1002/(SICI)1521-3773(20000515)39:10<1771::AID-ANIE1771>3.0.CO;2-D 10934356

[B53] Zhang F. Jin Y. Shi J. Zhong Y. Zhu W. El-Shall M. S. (2015a). Polyoxometalates confined in the mesoporous cages of metal–organic framework MIL-100(Fe): efficient heterogeneous catalysts for esterification and acetalization reactions. Chem. Eng. J. 269, 236–244. 10.1016/j.cej.2015.01.092

[B54] Zhang W. Meng T. Tang J. Zhuang W. Zhou Y. Wang J. (2017). Direct synthesis of 2,5-diformylfuran from carbohydrates using high-silica MOR zeolite-supported isolated vanadium species. ACS Sustain. Chem. Eng. 5, 10029–10037. 10.1021/acssuschemeng.7b02002

[B55] Zhang X. Wilson K. Lee A. F. (2016). Heterogeneously catalyzed hydrothermal processing of C_5_-C_6_ sugars. Chem. Rev. 116, 12328–12368. 10.1021/acs.chemrev.6b00311 27680093

[B56] Zhang Z. M. Zhang T. Wang C. Lin Z. Long L. S. Lin W. (2015b). Photosensitizing metal-organic framework enabling visible-light-driven proton reduction by a Wells-Dawson-type polyoxometalate. J. Am. Chem. Soc. 137, 3197–3200. 10.1021/jacs.5b00075 25712689

[B57] Zhao M. Yuan K. Wang Y. Li G. Guo J. Gu L. (2016). Metal-organic frameworks as selectivity regulators for hydrogenation reactions. Nature 539:76 10.1038/nature19763 27706142

[B58] Zhou Y. Chen G. Long Z. Wang J. (2014). Recent advances in polyoxometalate-based heterogeneous catalytic materials for liquid-phase organic transformations. RSC Adv. 4, 42092–42113. 10.1039/C4RA05175K

