# Supplementary material for: Synergistic Catalysis of Ruthenium Nanoparticles and Polyoxometalate Integrated Within Single UiO−66 Microcrystals for Boosting the Efficiency of Methyl Levulinate to γ-Valerolactone
Source: Front Chem. 2019 Feb 1;7:42. doi: 10.3389/fchem.2019.00042 (PMC6367244; doi:10.3389/fchem.2019.00042)
Supplement: Supplementary file 1 [file Data_Sheet_1.doc]

Supporting Information

Synergistic Catalysis of Ruthenium Nanoparticles and Polyoxometalate Integrated within Single UiO–66 Microcrystals for Boosting the Efficiency of Methyl Levulinate to γ-Valerolactone

Xiaoxiong Cai, Qionghao Xu, Gaomei Tu*, Yanghe Fu, Fumin Zhang*, Weidong Zhu*

Key Laboratory of the Ministry of Education for Advanced Catalysis Materials, Institute of Physical Chemistry, Zhejiang Normal University, 321004 Jinhua, People's Republic of China

*Corresponding Authors: E-mail: tugaomei@zjnu.cn; zhangfumin@zjnu.edu.cn; weidongzhu@zjnu.cn

1. **Chemicals**

Zirconium (IV) chloride (ZrCl_4_, 98%), tungstosilicic acid hydrate (H_4_SiW_12_O_40_[**·xH_2_O**](https://baike.sogou.com/lemma/ShowInnerLink.htm?lemmaId=7656578&ss_c=ssc.citiao.link), abbreviated as SiW), ruthenium (III) chloride (RuCl_3_, 43−44%), and γ-valerolactone (GVL, 99%) were purchased from Aladdin Industrial Inc; acetic acid (≥99.5%), N,N-dimethylformamide (DMF, ≥99.5%), ethylene glycol (EG, ≥99.0%), methanol (≥99.5%), ethanol (≥99.7%) and acetone (≥99.5%) were obtained from Sinopharm Chemical Reagent Co., Ltd; terephthalic acid (H_2_BDC, >99%) and methyl levulinate (ML, >99%) were obtained from TCI; polyvinyl pyrrolidone (PVP, M_w_ = 58000, ≥99.0%) was obtained from Xiya Reagent Co., Ltd., and H_2_ (99.999%) and Ar (99.999%) were purchased from Shanghai Pujiang Special Gas Co., Ltd. All commercial chemicals were used without further purification.

1. Characterization

X-ray powder diffraction (XRD) patterns of the various samples were recorded on a Philips PW3040/60 diffractometer with Cu *Kα* radiation, which was operated at 40 kV, 40 mA, and *λ* =0.1542 nm.

N_2_ adsorption isotherms were obtained at -196 °C on a Micromeritics ASAP 2020 instrument. Based on N_2_ adsorption, the Brunauer–Emmett–Teller (BET) specific surface area (*S_BET_*) was calculated by using the multiple-point BET method in the relative pressure range of P/P_0_ = 0.05–0.25. The pore volume (*V_micro_*) was determined by the t-plot method.

Fourier transformed infrared (FTIR) spectra were collected on a Nicolet NEXUS670 infrared spectrometer, with the catalyst powder mixed with KBr, and subsequently pressed onto a self-supporting wafer.

Thermogravimetric analysis (TGA) was performed on a Rigaku Thermo plus EVO2 thermal analysis system with the use of nitrogen flow (40 mL/min) and a heating rate of 10 °C/min in the range of 25–800 °C.

X-ray photoelectron spectroscopy (XPS) measurements were performed on a Thermo VG ESCALAB250 system with a base pressure of 10^−9^ Torr. The XPS data were internally calibrated by setting the binding energy of C 1s at 284.6 eV.

The Ru contents of the various catalysts were examined by inductively coupled plasma–atomic emission spectrometer (ICP–AES) on an IRIS Intrepid II XPS instrument.

Scanning electron microscopic (SEM) analyses were performed on a Hitachi S–4800 apparatus. Transmission electron microscopy (TEM) images were acquired on a JEOL JEM–1200 apparatus. The samples for TEM were prepared by dispersing them in ethanol, and by dropping them in a perforated carbon-coated copper grid. Besides, energy-dispersive X-ray spectroscopy (EDS) elemental mapping analyses were also performed to verified the presence and the distribution of Si, W and Ru NPs.

The acid capacity of SiW@UiO–66 was determined by acid–base titration using NaCl solution as an ion-exchange agent. Typically, 0.05 g of the pre-dried SiW@UiO–66 was suspended in 60 mL of 0.01 mol/L aqueous NaCl solution. The resulting suspension was gently stirred for 24 h at room temperature to achieve a thorough cation exchange between the acidic proton of SiW and Na^+^ from the solution. Subsequently, the suspension was filtrated and the filtrate was titrated with a 0.1 mol/L NaOH solution to quantify the exchanged acidic proton, as described above.

**Table S1** Representative data for catalytic conversion of ML to GVL by heterogeneous catalysts compared with our work

| Catalyst | s/c^a^ | solvent | Temp  (°C) | *p*_H2_^b^  (MPa) | *t*  (min) | Con.^c^  (%) | Sel.^d^  (%) | Reference |
| --- | --- | --- | --- | --- | --- | --- | --- | --- |
| 2.0% Ru/11.7% SiW@UiO-66 | 200 | H_2_O | 80 | 0.5 | 120 | 94.1 | 74 | This work |
| 5.0% Ru/C | 348 | CH_3_OH | 130 | 1.2 | 160 | 97.8 | 89.4 | Green. Chem., 2012, 14, 1260-1263. |
| 5.0% Ru/C | 118 | CH_3_OH | 130 | 3.5 | 120 | 95 | 91 | Appl. Catal., A, 2014, 470, 215-220 |
| 4.5% Ru/Zr5SMS | 199 | CH_3_OH | 70 | 0.5 | 240 | >99.9 | 67.1 | Catal. Today,  2015, 258, 262–269. |
| 5.0% Ru/SO_3_H-UiO-66 | 200 | H_2_O | 70 | 0.5 | 240 | >99.9 | 74.5 | RSC Adv., 2017, 7, 44082-44088 |
| 2.0% Ru/SO_3_H-MIL-101 | 200 | H_2_O | 80 | 0.5 | 120 | 95.3 | 68.2 | Appl. Catal. A, 2018, 563 54-63. |

a: Molar ratio of ML to Pd in the catalyst used. b: H_2_ pressure. C: Conversion of ML. d: Selectivity for GVL.

Figure S1. TGA curves of the synthesized UiO-66, 11.7% SiW@UiO-66, 2.0% Ru/11.7% SiW@UiO-66 and the spent 2.0% Ru/11.7% SiW@UiO-66.


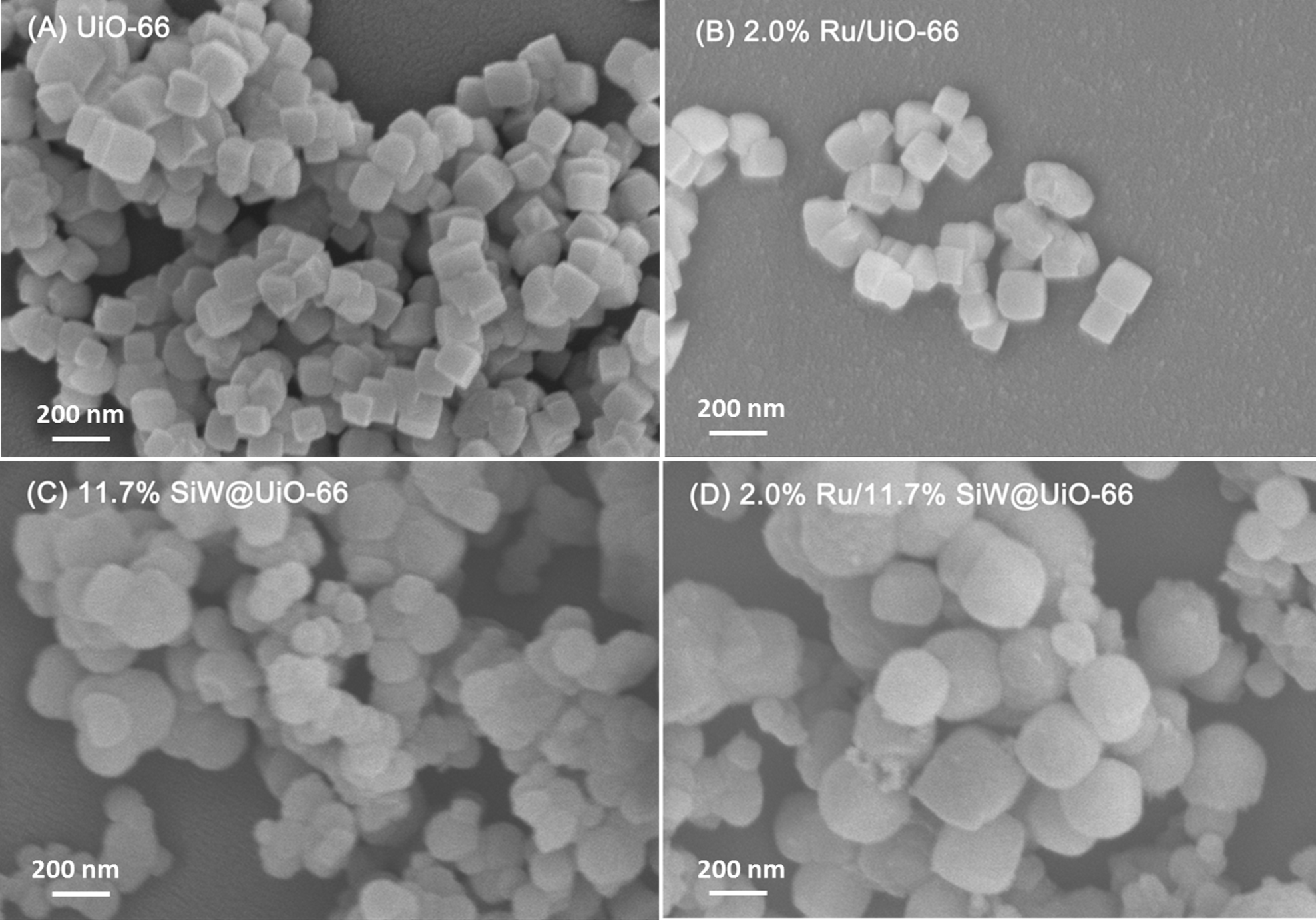


Figure S2. SEM image of the synthesized UiO-66


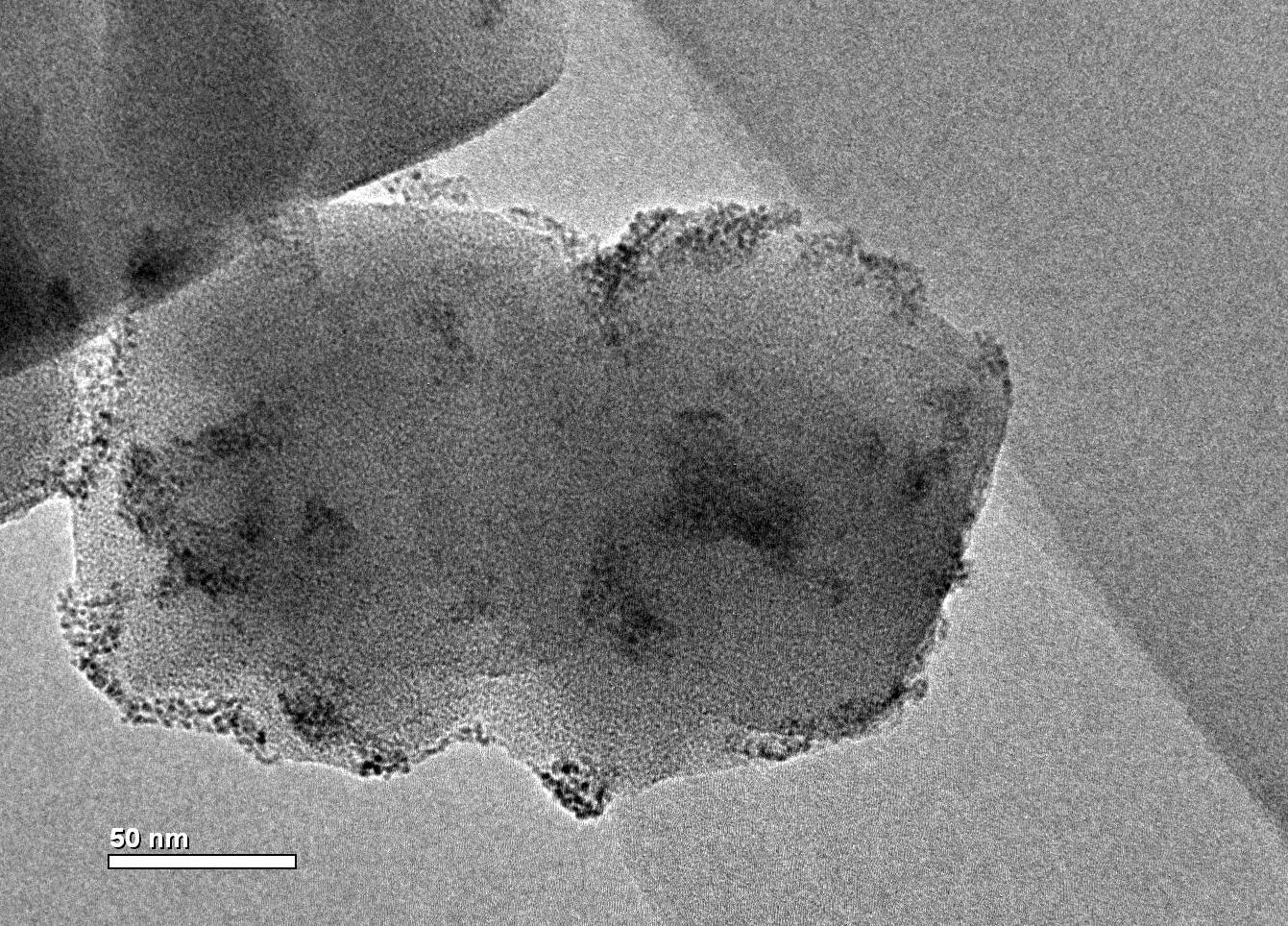


Figure S3. TEM image of 2.0% Ru/UiO-66.


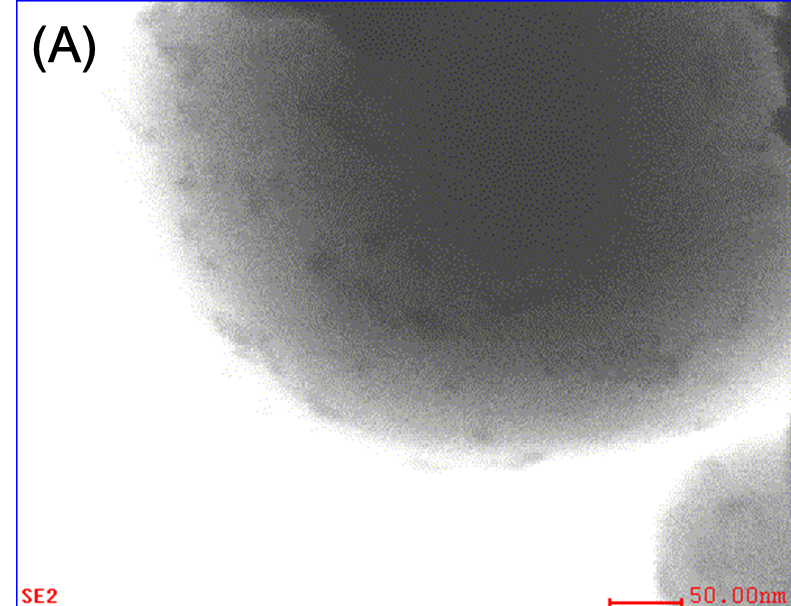

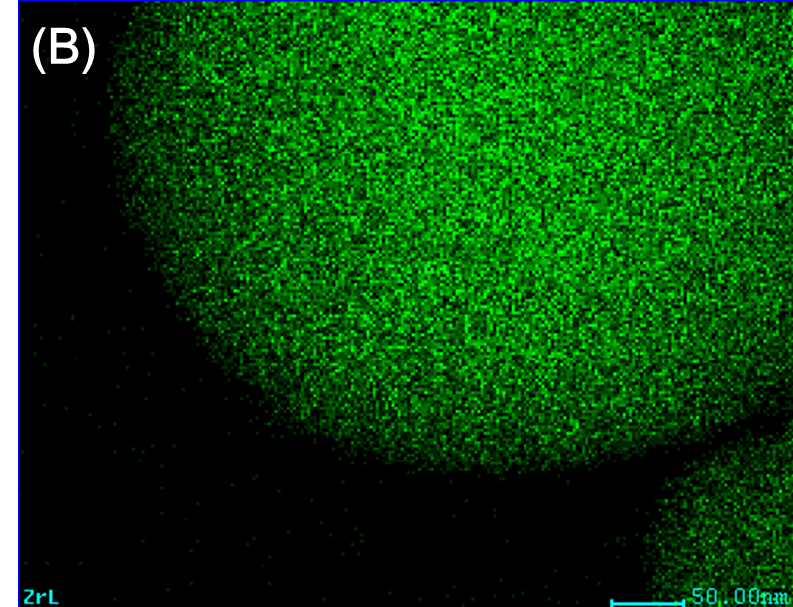


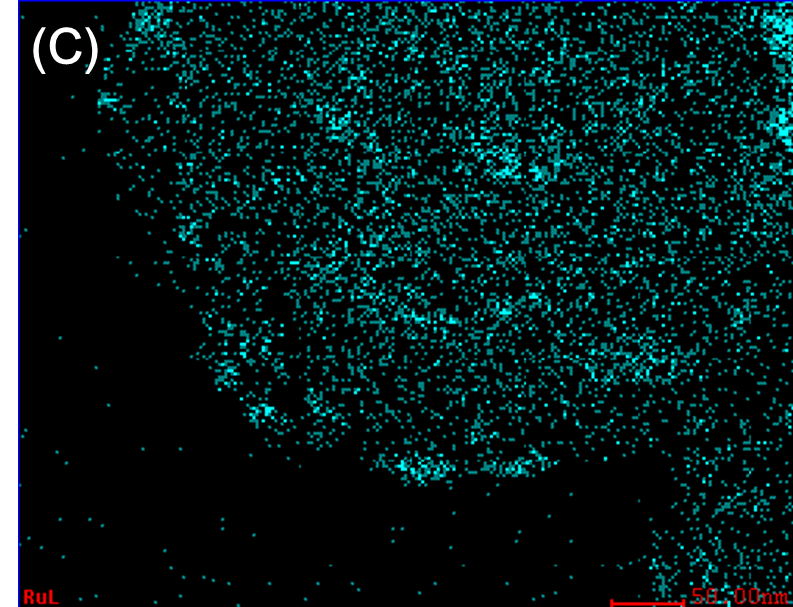

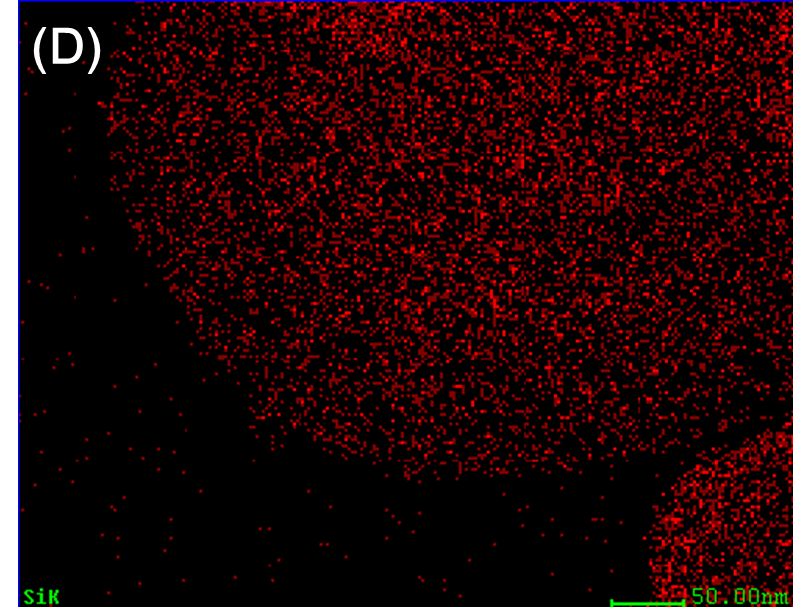


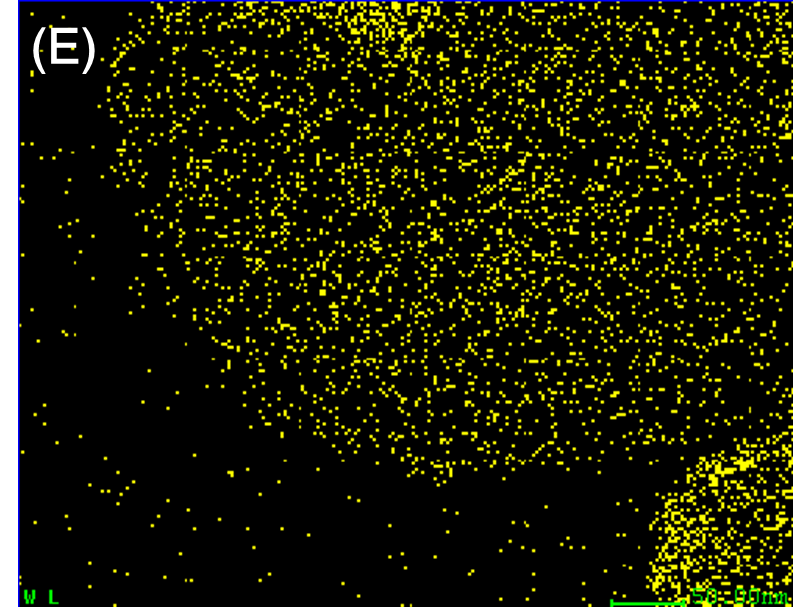


Figure S4. TEM image and EDX elemental maps of 2.0% Ru/11.7% SiW@UiO-66.

Figure S5. Concentration evolution profiles of ML, MHV, and GVL versus time for 2.0% Ru/UiO–66 at 80 °C. Reaction conditions: 0.257 g of ML, 50 mg of catalyst, 15 mL of H_2_O, and a H_2_ pressure of 0.5 MPa.

Figure S6. Concentration evolution profiles of ML, MHV, and GVL versus time for catalyst (5 mg of SiW + 50 mg of 2.0 % Ru/UiO–66) at 80 °C. Reaction conditions: 0.257 g of ML, 15 mL of H_2_O, and a H_2_ pressure of 0.5 MPa.

Figure S7. High-resolution XPS spectra of Zr (A), Si (B), W (C) and Ru (D) for the recycled 2.0% Ru/11.7% SiW@UiO-66 catalyst.


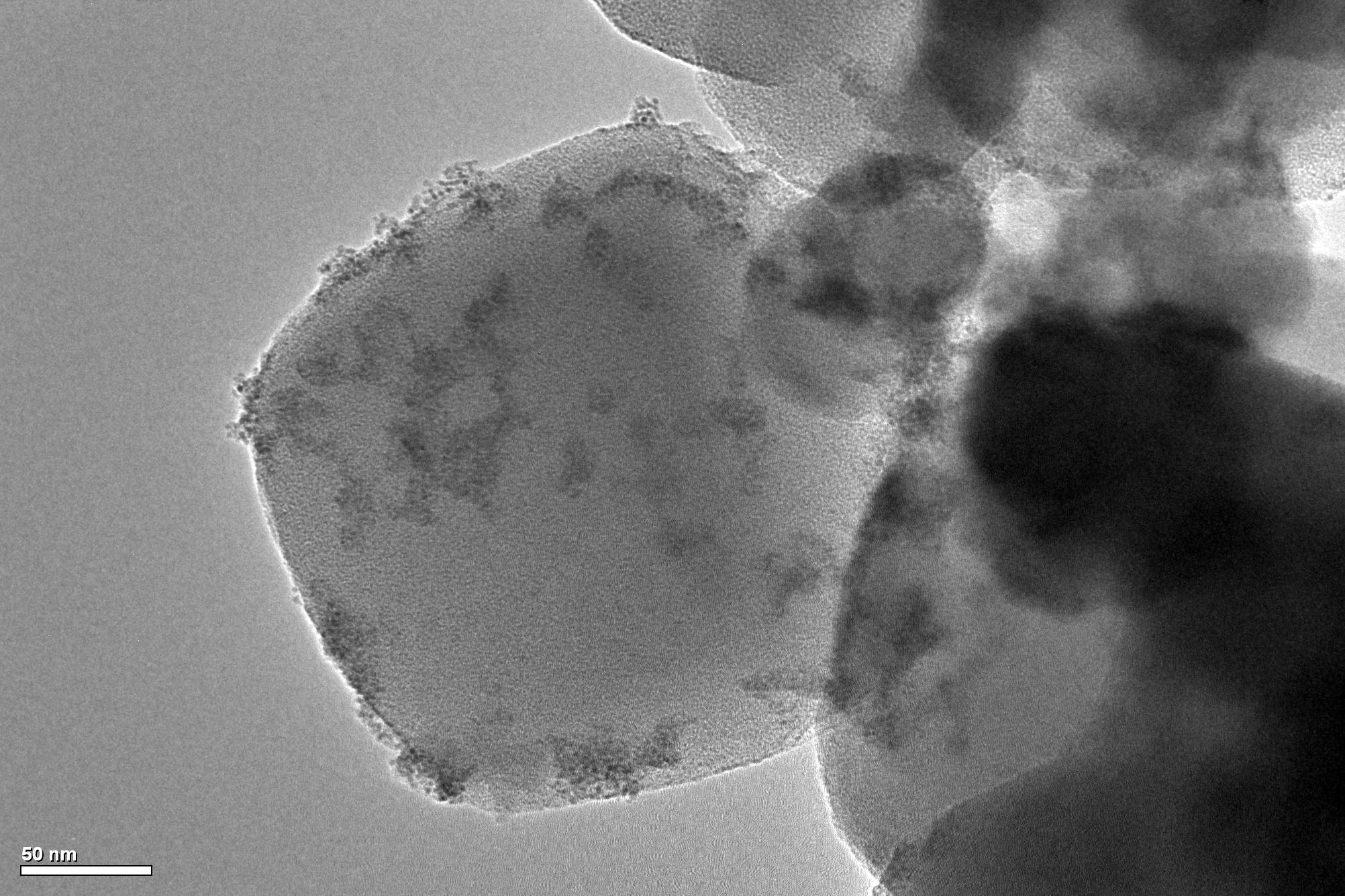


Figure S8. TEM image of the recycled 2.0% Ru/11.7% SiW@UiO-66 catalyst.
